# Does cognitive function impairment affect the duration of hospitalization and in-hospital mortality in geriatric patients hospitalized for COVID-19?

**DOI:** 10.1371/journal.pone.0284977

**Published:** 2023-04-25

**Authors:** Jarosław Janc, Anna Woźniak, Patrycja Leśnik, Lidia Łysenko

**Affiliations:** 1 Department of Anesthesiology and Intensive Therapy, 4th Military Clinical Hospital, Wroclaw, Poland; 2 Department of Anesthesiology and Intensive Therapy, Wroclaw Medical University, Wroclaw, Poland; Instituto Nacional de Geriatria, MEXICO

## Abstract

**Aims:**

To assess the effect of cognitive function, performance of activities of daily living (ADLs), degree of depression, and fear of infection among geriatric patients hospitalized in internal medicine wards for COVID-19 on the duration of hospitalization and in-hospital mortality.

**Methods:**

This observational survey study was conducted during the second, third, and fourth waves of the COVID-19 pandemic. The study included elderly patients of both sexes, aged ≥ 65 years, hospitalized for COVID-19 in internal medicine wards. The following survey tools were used: AMTS, FCV-19S, Lawton IADL, Katz ADL, and GDS15. The duration of hospitalization and in-hospital mortality were also assessed.

**Results:**

A total of 219 patients were included in the study. The results showed that impaired cognitive function in geriatric patients (AMTS) was associated with higher in-hospital mortality among COVID-19 patients. There was no statistical significance between fear of infection (FCV-19S) and risk of death. The impaired ability to perform complex ADLs (Lawton IADL) before the onset of the disease was not associated with higher in-hospital mortality among COVID-19 patients. The diminished ability to perform basic ADLs (Katz ADL) before the onset of the disease was not associated with higher in-hospital mortality in COVID-19. The degree of depression (GDS15) was not associated with higher in-hospital mortality in COVID-19 patients. Statistically, significantly better survival was observed for patients with normal cognitive function (p = 0.005). No statistically significant differences in survival were observed in relation to the degree of depression or independence in performing ADLs. Cox proportional hazards regression analysis showed a statistically significant effect of age on mortality (p = 0.004, HR 1.07).

**Conclusion:**

In this study, we observe that cognitive function impairments and the older age of patients treated for COVID-19 in the medical ward increase the in-hospital risk of death.

## Introduction

From the first half of the 14th century to 2018, 22 local or global epidemics were documented [1]. The latest Coronavirus Disease 2019 (COVID-19) pandemic was declared by the World Health Organization (WHO) on March 11, 2020. According to WHO data, as of December 14, 2022, 646,266,987 people in the world contracted COVID-19, of whom 6,636,278 died [2].

Based on analyses of previous epidemics, there are already many insights into “pandemic psychology,” and recognizing psychosocial aspects can play a key role in prevention efforts and the public health context [3]. Among the psychological aspects accompanying the pandemic, the occurrence of anxiety, distrust, denial of illness, isolation, and loneliness are emphasized. There may also be inappropriate defensive reactions (e.g., stigmatization and exclusion) [4, 5]. A large cohort study by Taquet et al. [6] found that the frequency of diagnosis of mental disorders between 14 and 90 days after recovery from COVID-19 was 18.1% and in 5.8% of cases it was the first diagnosis of mental problems. These values far exceed the incidence of mental disorders in patients with influenza or other respiratory diseases.

In the context of the COVID-19 pandemic, attempts have already been made to present its psychosocial consequences from the point of view of various social groups. Literature analysis concerns the risk or occurrence of specific disorders in adult patients, such as overload, decompensation, stress reactions, existential anxiety, addictions, eating disorders, reduced social activity, social withdrawal, and social anxiety caused by fear of infection, or exacerbation of already existing psychiatric disorders [7, 8]. In geriatric patients, loneliness, depression, and fear of COVID-19 infection are mentioned [9]. In the context of psychosocial aspects of the pandemic, there is a vicious circle in which the infectious disease has social and individual stress-related effects, with stress drastically reducing immunity and thus increasing susceptibility to the infectious disease [10], resulting in all possible consequences—longer duration of hospitalization or higher mortality [11, 12].

The study sought to answer the question of whether the cognitive function, performance of activities of daily living, degree of depression, and fear of infection in a group of geriatric patients hospitalized in internal medicine wards for COVID-19 had an impact on the duration of hospitalization and in-hospital mortality.

## Methods

### Study group

A multicenter, prospective, observational survey study was conducted between November 2020 and March 2022 during the second, third, and fourth waves of the COVID-19 pandemic. The study included elderly (geriatric) patients of both sexes, aged ≥ 65 years, hospitalized for COVID-19 in internal medicine wards, who gave informed consent to participate in the study.

### Qualification procedure

Inclusion criteria were as follows: a positive test result for SARS-CoV-2, hospitalization for SARS-CoV-2 infection, age equal to or over 65, the ability to obtain informed consent from the patient, and the ability to complete surveys. Exclusion criteria were as follows: age under 65, lack of informed consent, no SARS-CoV-2 infection, and previously diagnosed depressive disorders treated before hospitalization, delirium, severe cognitive impairment, unprotected hearing loss or lack of corrective glasses.

### Ethical considerations

The study protocol was approved by the Bioethics Committee of the Military Medical Chamber on 21 Jan 2022 in Poland (permission no. 191/22). The study was conducted in accordance with the guidelines of the Declaration of Helsinki and Good Clinical Practice. Written informed consent was obtained from all patients prior to study participation. The Strengthening the Reporting of Observational Studies in Epidemiology (STROBE) guidelines were followed due the observational protocol of this study.

### Research tools

The study was conducted once directly at the patient’s bedside using validated survey tools. These included the Abbreviated Mental Test Score (AMTS), the Fear of COVID-19 Scale (FCV-19S), the Lawton Instrumental Activities of Daily Living (IADL) Scale, the Katz Index of Independence in Activities of Daily Living (Katz ADL) and the 15-item Geriatric Depression Scale (GDS15). The duration of hospitalization and in-hospital mortality were assessed. All research tools used were validated in Polish and standardized for use in hospitalized patients of both sexes [13–16].

#### Abbreviated mental test score (AMTS)

The AMTS consists of 10 questions on personal data (the patient’s age, date of birth, home address), stating the time to the nearest hour, recognizing two persons (doctor, nurse), identifying the current year, naming the president of the country in which one lives, stating the year of the beginning of the Second World War, memorizing and recalling a fictitious address, and counting backwards by 1, from 20 to 1. In this test, the subject receives 1 point for each correct answer or 0 for no or wrong answer. The score ranges from 0 to 10 points, with 0–3 points indicating severe memory impairment, 4–6 points indicating moderate memory impairment, 7–8 points indicating mild memory impairment, and 9–10 points indicating normal status [17]. The Polish modified version of AMTS has been included as an element of overall geriatric assessment aught as part of continued training for medical staff, and in geriatric care standards in Poland [13].

#### Fear of COVID-19 Scale (FCV-19S)

The FCV-19S contains seven statements that evaluate the patient’s own feelings. They concern the following: fear of being infected with SARS-CoV-2, anxiety when thinking about COVID-19, clammy hands at the thought of COVID-19, fear of losing one’s life due to COVID-19, nervousness and anxiety when following information about COVID-19 in the media, insomnia when thinking about the threat of COVID-19, accelerated heart rate, or uneven heartbeat when thinking about the risk of contracting COVID-19. The patient responses are measured on a 5-point scale, from 1 point (strongly disagree) to 5 points (strongly agree). The range of results is between 7 and 35 points; a higher total score indicates a higher intensity of fear of COVID-19 infection [18]. The FCV-19S refers to feelings before infection. Patients participating in the study were informed in detail before its completion. The scale was validated in Polish [14].

#### Lawton instrumental activities of daily living (IADL) scale

The IADL is an instrument used to assess more complex independent living skills. This scale contains eight questions concerning the ability to perform the following tasks independently: using the telephone, using public transportation, shopping for groceries, preparing meals, performing household chores (e.g., cleaning), doing laundry, handling medications, and handling finances. Points from 1 to 3 are awarded for the answer to each question, with 1 point indicating lack of independence, 2 points indicating moderate independence, and 3 points indicating full independence. The maximum number of points on this scale is 24, indicating proficiency in the performance of complex activities of daily living [19]. The IADL scale was validated in Polish [15].

#### Katz index of independence in activities of daily living (Katz ADL)

Katz ADL is one of the most popular methods for assessing functional status in a group of elderly people. It is a scoring system that awards 1 point or 0, depending on the degree of function. Points are awarded in six basic areas of independence: full-body bathing in a bathtub or shower, dressing, toileting, transferring from bed to chair and back again, feeding, and continence. The results are presented in the form of three ranges: full function (5–6 points), moderate impairment (3–4 points), and complete impairment (1–2 points) [20]. The Katz ADL scale was validated in Polish [15].

#### Geriatric depression scale, 15-item version (GDS15)

The GDS15 is a screening tool specifically designed to assess depression in the elderly. It contains 15 simple questions to be answered yes or no, and, depending on the content of the question, 1 point or 0 is awarded for the answer. The maximum number of points obtained is 15, and the minimum is 0, with a score of 0–5 points indicating no depression, 6–10 points indicating moderate depression, and 11–15 points indicating severe depression [21]. The GDS15 scale was validated in Polish [16].

### Statistical analysis

Statistical analysis was performed using Statistica 13.1 software (TIBCO Software Inc., Palo Alto, USA). Means, medians, quartiles, minimum and maximum values, and standard deviations were calculated for measurable variables. All the studied quantitative variables were tested with the Shapiro-Wilk test to determine the type of distribution. Intergroup comparison of the results of quantitative variables was conducted using the t-test or the Mann-Whitney U test (depending on the fulfillment of the assumptions). The chi-squared test or Fisher’s exact test was used to compare qualitative variables. To perform the survival analysis, the Kaplan-Meier survival function estimation procedure was used based on continuous survival times. A comparison of survival in groups was performed with the log-rank test. The Cox proportional hazards model was used to assess the effect of selected variables on mortality. A level of α = 0.05 was assumed for all comparisons.

## Results

### Sociodemographic characteristics and hospital mortality

**Fig 1:**
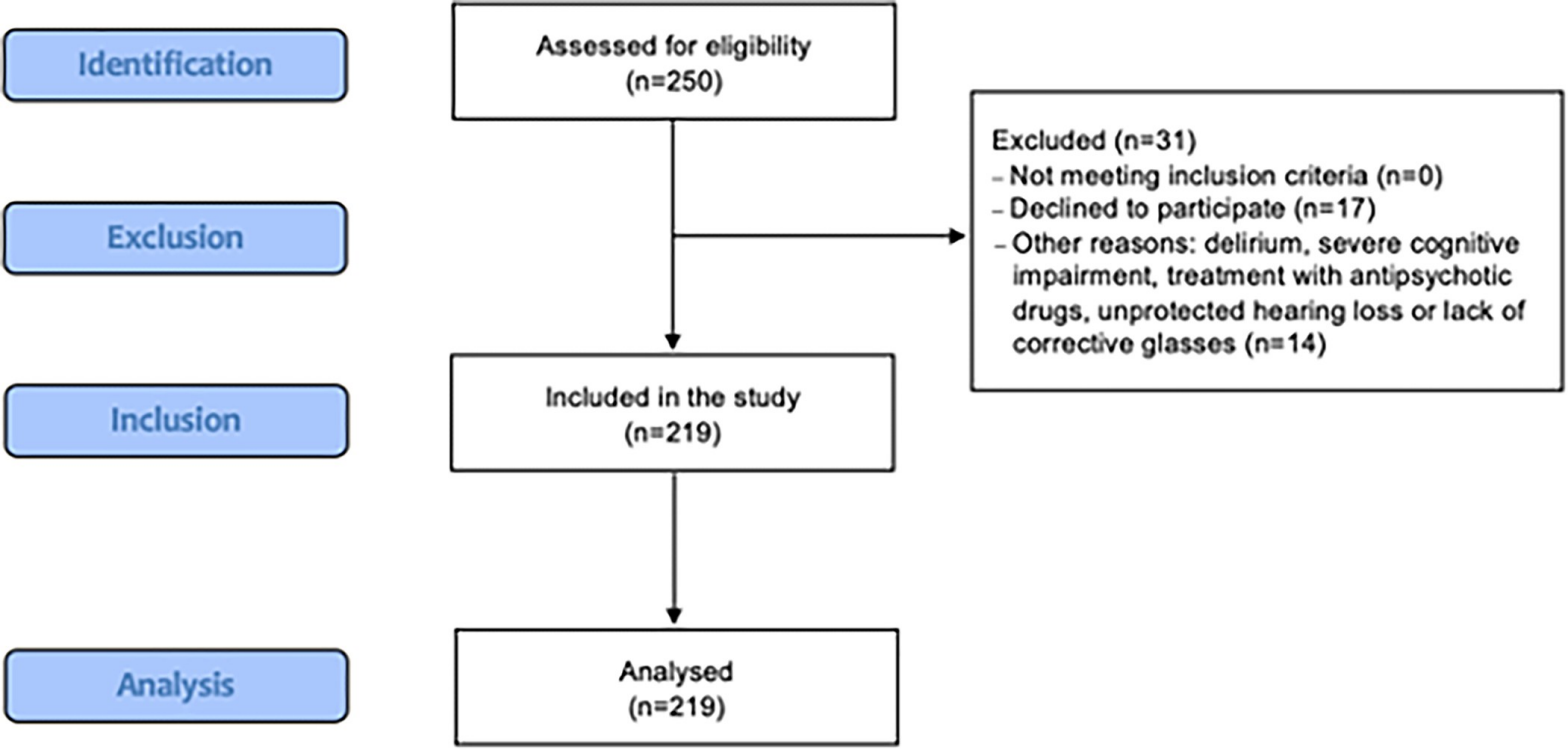
STROBE flow chart of study participants.

During the study period from November 2020 to March 2022, 250 patients were assessed for eligibility. Of these, 31 were excluded due they declined to participate in the study (n = 17) or had incomplete medical records (n = 14). Finally, a group of 219 patients were included in the study (Fig 1). The characteristics of the patients, stratified based on hospital mortality, are shown in Table 1. The total mortality rate during hospitalization in the analyzed population was 15.5%. Statistically significant differences in age in relation to hospital mortality were demonstrated (p <0.002).

**Table 1:** Characteristics of groups due to in-hospital mortality.

Variable		Group (n = 219)	In-Hospital mortality		p-value*
			No (n = 185)	Yes (n = 34)	
Sex	female	n = 128 (58%)	n = 106 (57%)	n = 22 (65%)	0.420
	male	n = 91(42%)	n = 79 (43%)	n = 12 (35%)	
Age	M±SD	78.2±8.4	77.4±8.2	82.4±8.3	**0.002**
	Me	78	76	84	
	Min-max	65–97	65–97	65–97	
Days of hospital stay	M±SD	17.8±11.3	18.2±11.9	15.9±7.6	0.585
	Me	15	15	14.5	
	Min-max	2–106	3–106	2–31	

Chi-squared or Fisher’s exact test for qualitative variables were used. N, number of patients; M, mean; SD, standard deviation; Me, median; Min, minimum; Max, maximum.

### Functional impairment and hospital mortality

In the AMTS scale, a mean (M) of 9 points with interquartile range (IQR) 7–10, and min-max 0–10 was obtained, while normal function status was obtained in 126 patients (58%), mild memory impairment in 53 patients (24%), moderate memory impairment in 24 patients (11%), and severe memory impairment in 16 patients (7%). Detailed results are presented in Table 2.

**Table 2:** Descriptive statistics of particular variables.

Variables		Mean	SD	Median	Min	Max	Q1	Q3
Total score AMTS		8.0	2.3	9.0	0.0	10.0	7.0	10.0
FCV-19S		19.1	5.9	18.0	7.0	35.0	14.0	23.0
IADL		20.2	4.6	22.0	8.0	24.0	17.0	24.0
ADL		4.2	2.0	5.0	0.0	6.0	3.0	6.0
GDS15		4.1	3.1	4.0	0.0	14.0	2.0	6.0
AMTS	Severe impairment	n = 16 (7%)						
	Moderate impairment	n = 24 (11%)						
	Mild impairment	n = 53 (24%)						
	Normal functioning	n = 126 (58%)						
GDS15	No signs of depression	n = 156 (71%)						
	Moderate depression	n = 56 (26%)						
	Severe depression	n = 7 (3%)						
ADL	Functional	n = 127 (58%)						
	Moderate function	n = 45 (21%)						
	Not functional	n = 47 (21%)						

SD, standard deviation; Min, minimum; Max, maximum; Q1, lower quartile; Q3, upper quartile; n, number of patients; %, percentage of patients; AMTS, Abbreviated Mental Test Score; FCV-19S, Fear of COVID-19 Scale; IADL, Lawton Instrumental Activities of Daily Living Scale, Katz ADL, Katz Index of Independence in Activities of Daily Living; GDS15, 15-item Geriatric Depression Scale.

The ATMS scale scores were also analyzed by comparing them in patients who died during hospitalization (n = 34) and those who were discharged from the hospital (n = 185). The median values obtained for the total score were 9 points in the group of patients who died and 8 points in the group of patients who survived. The difference between the total score values in the former and latter groups was statistically significant (p = 0.001). Similarly, statistically significant differences (p = 0.004, chi-square test) between the two groups were noted in the scores presented as ranges: normal functioning, 12 (35%) versus 114 (61%); mild memory impairment, 11 (32%) versus 42 (23%); moderate memory impairment, 9 (27%) versus 15 (8%); and severe memory impairment, 2 (6%) versus 14 (8%), respectively. The results showed that impaired cognitive function in geriatric patients was associated with higher in-hospital mortality in COVID-19 patients. There was no statistical significance between the FCV-19S scores and the risk of death. Detailed results are presented in Table 3.

**Table 3:** Comparison of the results including patients’ survival.

Outcome		Death														p-value
		No (n = 185)							Yes (n = 34)							
		M	SD	Me	Min	Max	Q1	Q3	M	SD	Me	Min	Max	Q1	Q3	
Total score AMTS		8.2	2.3	9.0	0.0	10.0	7.0	10.0	7.1	2.3	8.0	0.0	10.0	5.0	9.0	0.001*
FCV-19S		18.9	5.7	18.0	7.0	35.0	14.0	22.0	20.3	7.0	21.5	7.0	35.0	14.0	25.0	0.138
IADL		20.5	4.4	22.0	8.0	24.0	19.0	24.0	18.6	5.1	19.0	8.0	24.0	15.0	24.0	0.073
ADL		4.3	1.9	5.0	0.0	6.0	3.0	6.0	3.6	2.4	4.0	0.0	6.0	1.0	6.0	0.145*
GDS15		3.9	3.0	3.0	0.0	14.0	1.0	6.0	4.9	3.2	4.0	0.0	14.0	2.0	7.0	0.101*
AMTS	S	n = 14 (8%)							n = 2 (6%)							0.004**
	MT	n = 15 (8%)							n = 9 (27%)							
	MD	n = 42 (23%)							n = 11 (32%)							
	NF	n = 114 (61%)							n = 12 (35%)							
GDS15	NSD	n = 134 (72%)							n = 22 (65%)							0.120**
	MD	n = 47 (25%)							n = 9 (26%)							
	SD	n = 4 (2%)							n = 3 (8%)							
ADL	F	n = 111 (60%)							n = 16 (47%)							0.101**
	MF	n = 39 (21%)							n = 6 (18%)							
	NF	n = 35 (19%)							n = 12 (35%)							

* Mann-Whitney U or ** chi-square test were used. M, Mean; SD, standard deviation; Me, median; Min, minimum; Max, maximum; Q1, lower quartile; Q3, upper quartile; n, number of patients; %, percentage of patients; AMTS, Abbreviated Mental Test Score; FCV-19S, Fear of COVID-19 Scale; IADL, Lawton Instrumental Activities of Daily Living Scale, Katz ADL, Katz Index of Independence in Activities of Daily Living; GDS15, 15-item Geriatric Depression Scale; S, Severe; MT, Moderate; MD, Mild; NF, Normal Functioning; NSD, No Signs of Depression; MD, Moderate Depression; SD, Severe Depression; F, Functional; MF, Moderate Functional; NF, Not Functional.

The study showed that the impaired ability to perform complex activities of daily living using the Lawton IADL before the onset of the disease in the group of geriatric patients was not associated with higher in-hospital mortality in COVID-19. The diminished ability to perform the basic activities of daily living using Katz ADL before the onset of the disease was not associated with higher in-hospital mortality in COVID-19. No depression status was demonstrated in 156 patients (71%), moderate depression in 56 patients (26%), and severe depression in 7 patients (3%) using GDS15. The analysis showed that, in the group of geriatric patients, the degree of depression was not associated with higher in-hospital mortality in COVID-19.

### Assessment of the impact of selected variables on in-hospital survival

**Fig 2:**
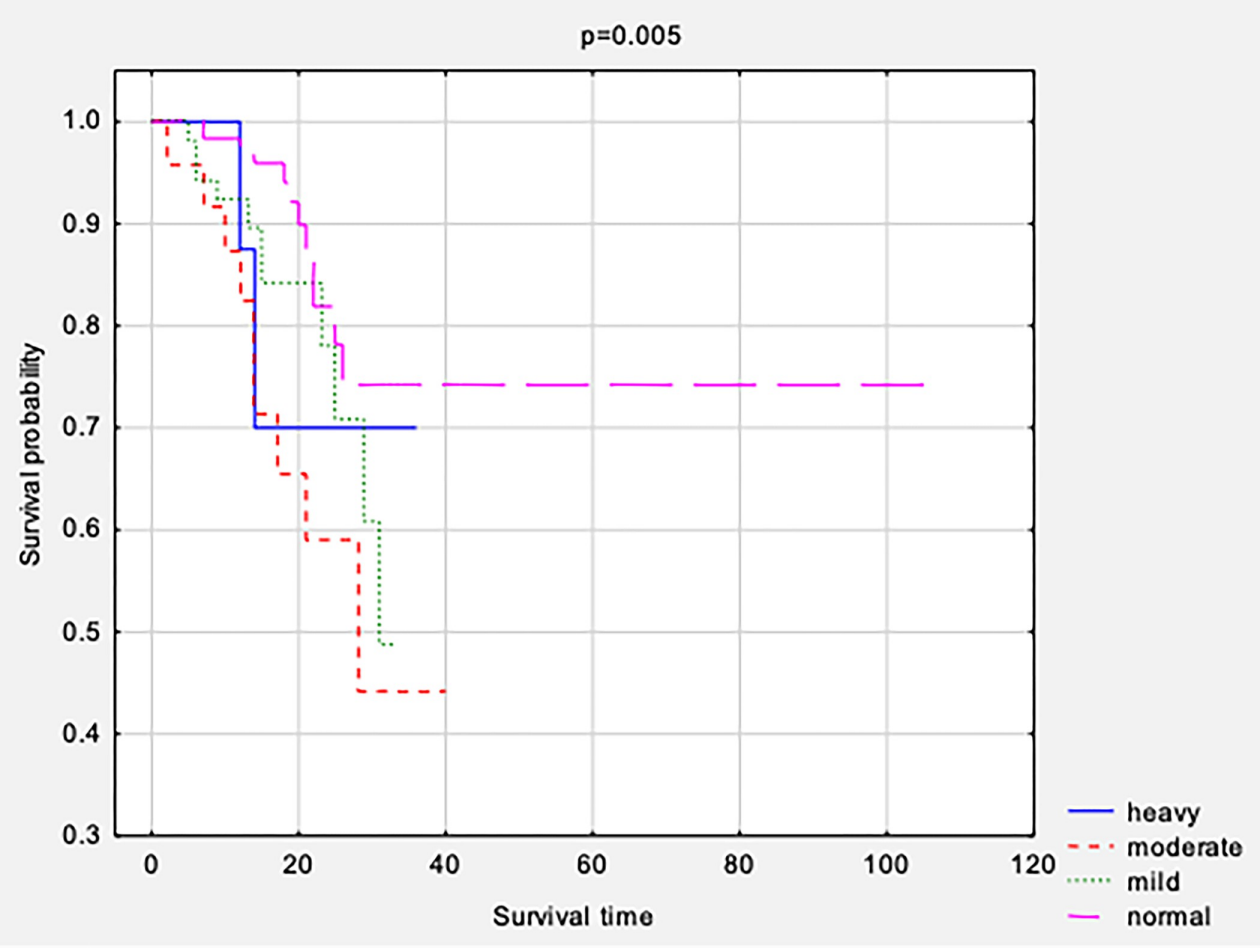
Kaplan-Meier curve showing the comparison of survival time according to abbreviated mental test score (AMTS).

**Fig 3:**
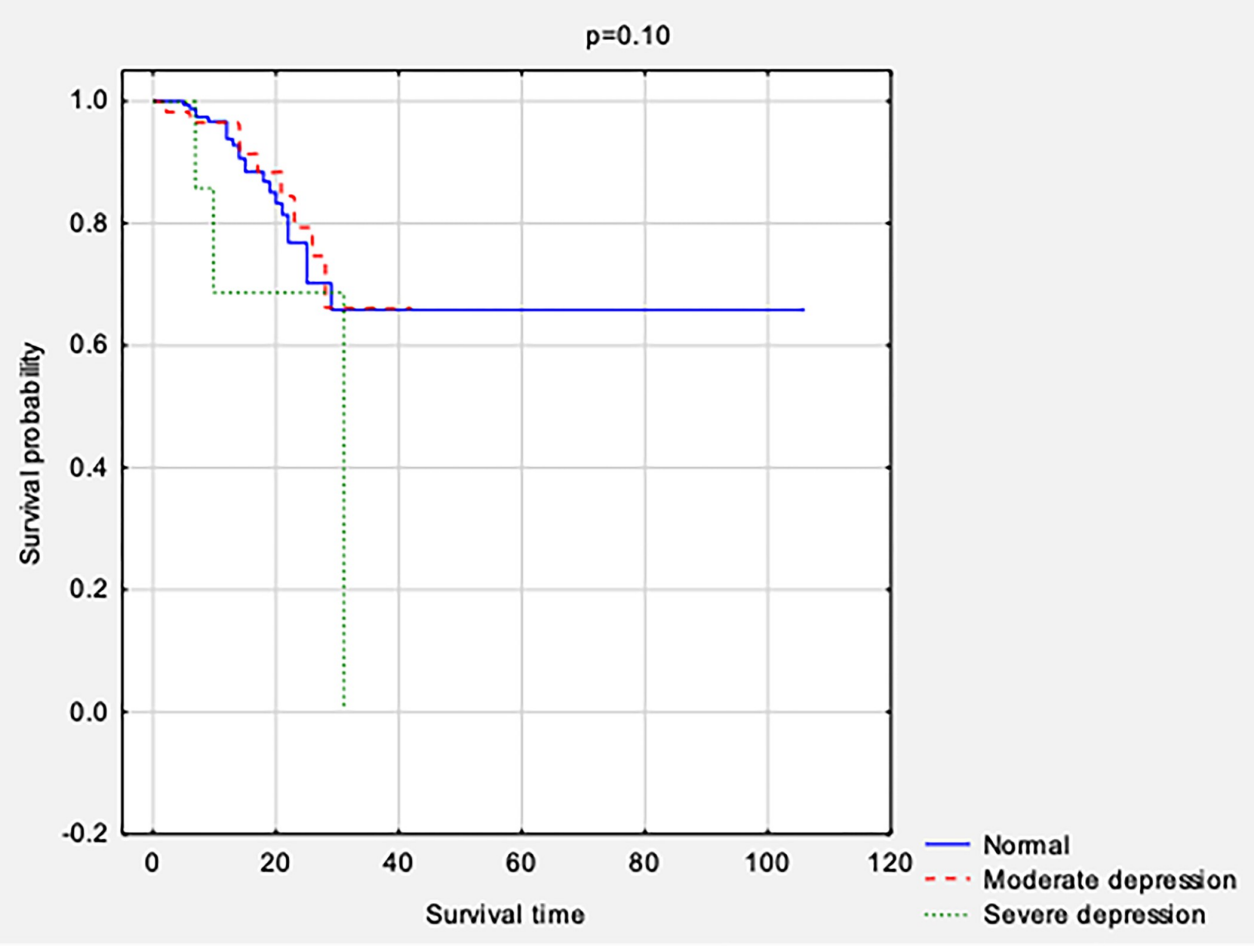
Kaplan-Meier curve showing a comparison of survival time according to the degree of depression (GDS15).

**Fig 4:**
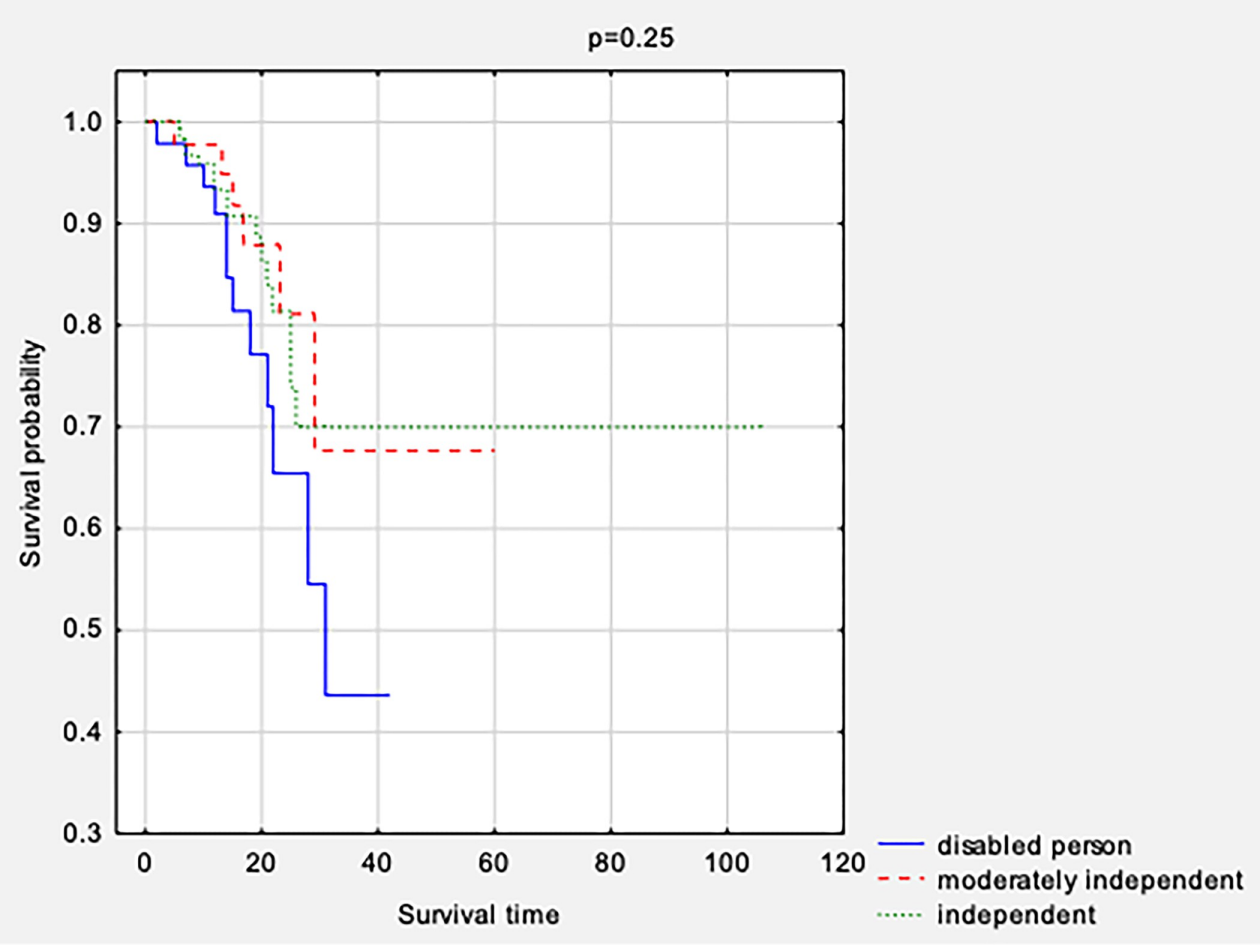
Kaplan-Meier curve showing a comparison of survival time according to independence in the performance of daily living activities (ADL).

The analysis of in-hospital survival time according to cognitive function, the degree of depression, or the ability to perform activities of daily living is presented in Kaplan-Meier survival curves (Figs 2–4). Statistically, significantly better survival was observed for those with normal cognitive function (Fig 2) (p = 0.005). No statistically significant differences in survival were observed in relation to the degree of depression (Fig 3) or independence in performing activities of daily living (Fig 4). Cox proportional hazards regression analysis showed a statistically significant effect of age on mortality (p = 0.004, HR 1.07) and cognitive function level (p = 0.006, HR 0.84). Detailed results are presented in Table 4.

**Table 4:** Assessment of the effect of variables on in-hospital mortality.

Variable		Parameter evaluation	Standard error	Chi-square	p-value	Relative Hazard (HR)	95% PU HR lower	95% PU HR upper
Age		**0.07**	**0.02**	**8.52**	**0.004**	**1.07**	**1.02**	**1.12**
Anxiety score		0.04	0.03	1.75	0.186	1.04	0.98	1.09
IADL		-0.05	0.03	2.83	0.092	0.95	0.89	1.01
ADL		-0.13	0.08	2.86	0.091	0.88	0.75	1.02
GDS15		0.05	0.05	0.84	0.359	1.05	0.95	1.17
AMTS		**-0.18**	**0.06**	**7.61**	**0.006**	**0.84**	**0.74**	**0.95**
Sex (ref. M)	F	0.18	0.18	0.97	0.325	1.43	0.70	2.89

* Cox proportional hazard regression, univariate model was used. M, male; F, female; AMTS, Abbreviated Mental Test Score; FCV-19S, Fear of COVID-19 Scale; IADL, Lawton Instrumental Activities of Daily Living Scale, Katz ADL, Katz Index of Independence in Activities of Daily Living; GDS15, 15-item Geriatric Depression Scale; HR, hazard ratio.

## Discussion

In the present study, the in-hospital mortality rate of the cohort of 219 patients hospitalized for COVID-19 in the internal ward was 15.5%. The study identified that the significant factors associated with higher in-hospital mortality were age and mental impairment assessed with the AMTS.

Mortality increased by 7% for each one-year increase in age. This relationship has also been reported in numerous studies. In a large cohort of hospitalized patients with COVID-19, Sundaram et al. [22] showed that increased age is an independent risk factor for mortality, with the odds risk approximately doubling for ages 65 to 79 compared to 50 and 64. In a 2022 systematic review, Dadras et al. [23] confirmed that COVID-19 mortality is independently associated with patient age, with older patients more likely to be admitted to intensive care and develop ARDS.

The present study demonstrated the impact of mental impairment on hospital mortality. Previously published studies have shown that diminished cognitive function, both in patients with COVID-19 [8, 9] and without COVID-19 (Thomas et al., 2019), is a strong risk factor for increased mortality. Cognitive dysfunction is often accompanied by disorders of other systems, including the gastrointestinal tract, with swallowing disorders, which increase the risk of aspiration pneumonia and malnutrition. All these factors contribute to increased mortality. A large systematic review by Thomas et al. (Thomas et al., 2013) that included 28 studies involving elderly hospitalized patients and 5 studies involving nursing home residents determined that the domains associated with mortality in a multivariable analysis in the highest proportions of studies are physical function, cognitive function, and nutrition for in-hospital mortality.

The present study found no effect of ADL impairment on mortality in the geriatric group, as described in the available publications [24, 25]. Only in-hospital mortality was assessed in the present study. Thomas et al. (Thomas et al., 2013) demonstrated the effect of physical function on mortality up to one year. It seems that the level of physical function mainly influences out-of-hospital survival when patients perform activities of daily living such as toileting, meal preparation, and shopping independently, without the medical staff and other assistance, which are often insufficient or unavailable. In the hospital setting, patients have their meals and medications prepared and receive physical therapy. A lack of support in daily life may lead to malnutrition, which is an independent predictor of mortality. As mentioned above, the large cohort study by Taquet et al. [6] found that the frequency of diagnosis of mental disorders between 14 and 90 days after recovery from COVID-19 was 18.1%, with the first diagnosis occurring in 5.8% of cases. These values far exceed the incidence of mental disorders in patients with influenza or other respiratory illnesses.

Clouston at al. showed that depression is a risk factor for mortality in COVID-19 patients. Atkins et al. [26] arrived at the same conclusion in 2020. Mechanisms may include the potential for neuro- and systemic immune dysfunction common among individuals with depression [27]. The present study found no effect of depressive disorders on mortality during hospitalization for COVID-19. However, higher levels of anxiety were observed in the female group than in the male group: M = 20 (IQR = 15–23, min-max = 7–35) vs. M = 16 (IRQ = 14–22; min-max = 7–35). Interestingly, women were significantly more likely than men to respond affirmatively to selected statements of the above scale: “I am (was) most afraid of being infected with the virus that causes COVID-19” (p = 0.004), “It makes (made) me uncomfortable to think about COVID-19” (p = 0.002), and “Watching and reading news and stories about COVID-19 in the media makes (made) me feel nervous or anxious” (p = 0.012).

Identifying the factors described above in the case of geriatric patients may help medical personnel identify patients with an increased risk of death. The challenge for future research is to determine whether qualitative prognostic information is sufficient in the clinical setting to rapidly reassess the benefits and harms of such elements of medical care as screening, prevention, and aggressive medical interventions.

## Conclusion

This study revealed that cognitive function impairments and the older age of patients treated for COVID-19 in the medical ward increase the in-hospital risk of death among geriatric patients.

## Study limitations

In the medical records hospitalized polish patients, among the patient’s personal data, question about level of education was not included. Hospitalized patients could reluctant to answer such a question, not seeing its purposefulness in the aspect of the treatment process. For the above reasons, such a question was not included in the research protocol. Also, future studies should consider assessing other outcomes potentially affecting the condition of older adults, such as frailty syndrome, especially in the context of geriatric patients with suspected unsuccessful aging.

## Supporting information

S1 Checklist STROBE statement—checklist of items that should be included in reports of *cross-sectional studies*. (DOCX)

S1 Data (CSV)

## Abbreviations

AMTS: Abbreviated Mental Test Score
COVID-19: Coronavirus Disease 2019
FCV-19S: Fear of COVID-19 Scale
GDS15: 15-item Geriatric Depression Scale
IADL: Lawton Instrumental Activities of Daily Living scale
IQR: interquartile range
Katz ADL: Katz Index of Independence in Activities of Daily Living
SARS-CoV-2: severe acute respiratory syndrome coronavirus 2
STROBE: Strengthening the Reporting of Observational Studies in Epidemiology guidelines
WHO: World Health Organization
